# Treatment effects of Herbst appliance in skeletal Class II cases during pre-pubertal and post-pubertal periods: a cone-beam computed tomographic study

**DOI:** 10.1038/s41598-023-37394-5

**Published:** 2023-07-13

**Authors:** Khaled Farouk, Osama Eissa, Ahmed Ghoneima, Ashraf El-Bedwehi, Ezzat Abdel-Fattah, Farouk Hussein, Tarek El-Bialy

**Affiliations:** 1 Department of Orthodontics, Faculty of Dental Medicine, Al-Azhar University, Cairo, Egypt; 2 Faculty of Medicine and Dentistry, 7-020D Katz Group Centre for Pharmacy and Health Research University of Alberta, Edmonton, Canada; 3 Department of Orthodontics, Faculty of Dentistry, Tanta University, Tanta, Egypt; 4 Department of Orthodontics and Oral Facial Genetics, School of Dentistry, Indiana University, Indianapolis, USA; 5 Department of Orthodontics and Pediatric Dentistry, Hamdan Bin Mohammed College of Dental Medicine (HBMCDM), Mohammed Bin Rashid University of Medicine and Health Sciences (MBRU), Dubai, UAE

**Keywords:** Health care, Health occupations, Medical research

## Abstract

In the present study, Thirty-six Class patients II (A condition in which the upper jaw is larger than the lower jaw) were randomly selected and assigned to one of two groups based on their maturation stage: the pre-pubertal group (18 patients, mean age 9.15 ± 1.5 years) and post-pubertal group (18 patients, mean age 16.3 ± 1.0 years). All patients were treated with a metallic splint-supported Herbst IV appliance (An appliance that acts like artificial joint working between the upper and power jaws that keeps the lower jaw in a forward position, thus improving the Class II condition). Pretreatment (T1) and post-Herbst IV treatment (T2) scans were obtained for both groups. Dental and skeletal measurements were made on the scans and statistically analyzed using paired and independent t-tests. The study hypothesis was that; the dentoskeletal changes in Class II malocclusion treatment using Herbst appliance in the Pre-pubertal is more than the Post-pubertal growth stage due to the remaining growth potential for the pre-pubertal patients. The comparison between the two groups revealed statistically significant differences in horizontal skeletal parameters in the lower jaw only, while other readings were similar.

## Introduction

One of the controversial topics in orthodontics is the best time to treat Class II malocclusions with mandibular retrognathism. During the pre-pubertal phase, with high growth potential, functional appliance treatment can alter the jaw relationship resulting in dentoalveolar and/or orthopedic changes^1–3^. Although the growth continues at a slower rate after the Pre-pubertal period, the post-adolescence Class II patients represent a large proportion of patients seeking orthodontic treatment mainly for aesthetic reasons. Numerous types of functional appliances have been advocated for the correction of Class II malocclusion with mandibular retrognathism. The main disadvantage of the removable functional devices is that extensive patient compliance is needed, and the treatment discontinuation rate can range between 9 and 15%^4^. The Herbst appliance is one of the most widely used fixed functional devices that is connected between the maxillary and mandibular dental arches, with the bilateral telescopic mechanism that keeps the mandible in a forward position^5^. There are several design variations of the Herbst appliance based on the attachment of telescopic mechanisms; banded, cast splint, stainless steel crowns, or acrylic resin splints. The Herbst IV is a further development of the standard Herbst appliance. Its ball hinge allows the patient greater freedom of lateral movement, increasing comfort and tolerance during treatment. Also, using the C-clips in Herbst IV is more convenient than the screws in the conventional Herbst.

It has been reported that the treatment of post-adolescent and young adults Class II cases with Herbst appliance could exploit the residual mandibular growth and induce remodeling processes in the region of the temporomandibular joint even after the age of 20 years and could consequently be a proper alternative to orthognathic surgery, particularly in mild skeletal Class II cases^6^.

The skeletal and dental effects of Herbst appliance in the pre-and post-pubertal periods had been investigated extensively by conventional cephalometric radiographs. However, identifying some landmarks such as condylar head, glenoid fossa, and TMJ spaces cannot be accurately detected using traditional cephalometric tracing/digitization techniques^3,7^.

The objective of this study was to compare the skeletal and dental effects of using cast splint supported Herbst IV appliance for the treatment of skeletal Class II malocclusion in the Pre-pubertal and Post-pubertal periods using CBCT. The null hypothesis was that the dentoskeletal changes in Class II malocclusion treatment using Herbst appliance in the Pre-pubertal is more than the Post-pubertal stage due to the remaining growth potential for the pre-pubertal patients, the alternate hypothesis was that there is no difference in the dentoskeletal changes in Class II malocclusion treatment using Herbst appliance in the Pre-pubertal and Post-pubertal growth stages.

## Results

The results of this study demonstrated significant dental effects in both groups. In the mandibular arch; forward movement of the lower incisors (pre-pubertal group: 4.08 mm ± 0.3, *P* = 0.01; post-pubertal group: 3.93 mm ± 0.2, *P* < 0.01), and lower molars (pre-pubertal group: 3.60 mm ± 0.1,* P* = 0.001; post-pubertal group: 2.89 mm ± 0.7, *P* = 0.002). In Maxillary arch, backward movement in the upper incisors (pre-pubertal group: − 1.03 mm ± 0.1;* P* = 0.02; post-pubertal group: − 1.48 mm ± 0.6,* P* = 0.03) and upper molars (pre-pubertal group: − 1.12 mm ± 0.5;* P* = 0.05; post-pubertal group: − 1.49 mm ± 0.6,* P* = 0.03) (Tables 1).

**Table 1:** Definitions of landmarks, 3D cephalometric planes.

Landmark	Definition
Sella (S)	The center of the hypophyseal fossa (sella turcica)
Nasion (N)	The midpoint of the frontonasal suture
Orbitale (Or)	The most inferior point of each infraorbital rim (right and left)
Glenoid fossa (G) (Fig. 6)	The deepest point in the glenoid (mandibular) fossa (right and left)
Porion (P) (Fig. 6)	The most superior point of each external acoustic meatus (right and left)
Point A	The point of maximum concavity in the midline of the alveolar process of the maxilla
Point B	The point of maximum concavity in the midline of the alveolar process of the mandible
Upper incisor (Ui)	The tip of the crown of the most protruded upper central incisor
Lower incisor (Li)	The tip of the crown of the most protruded lower central incisor
Upper molar (Um)	The point of greatest convexity in the mesial contour of the upper first molars (right and left)
Lower molar (Lm)	The point of greatest convexity in the mesial contour of the lower first molars (right and left)
3D cephalometric plane	
Frankfurt horizontal plane (FH)	Horizontal plane passes through the left Orbital and the two Porions (Pr–Pl) landmarks
Orbital perpendicular plane	Vertical plane passes through the right and left Orbitals (Orr–Orl) and perpendicular to the Frankfort horizontal Plane

**Table 2:** Paired t-test between the pre and post treatment CBCT readings in the pre and post- pubertal group.

Variable	Pre- pubertal group			Post- pubertal group		
	Pre- treatment (T1) Mean ± SD	Post-treatment (T2) Mean ± SD	*P*	Pre- treatment (T1) Mean ± SD	Post-treatment (T2) Mean ± SD	*P*
SNA	81.85° ± 1.8	81.63° ± 2.1	0.6	81.09° ± 2.1	80.44° ± 2.6	0.07
SNB	73.39° ± 1.7	74.92° ± 2.05	0.01*	74.15° ± 2.4	74.42° ± 2.7	0.2
Point A	13.27 mm ± 1.2	12.97 mm ± 1.5	0.4	13.73 mm ± 1.3	13.44 mm ± 1.4	0.3
Point B	2.9 mm ± 0.1	5,1 mm ± 0.5	0.003**	6.01 mm ± 0.4	6.5 mm ± 1.7	0.1
Glenoid fossa	62.47 mm ± 2.9	62.95 mm ± 3.1	0.6	65.25 mm ± 3.4	65.46 mm ± 3.4	0.6
Upper incisor	19.76 mm ± 2.1	18.73 mm ± 2.6	0.02*	22.18 mm ± 2.5	20.69 mm ± 2.1	0.03*
Lower incisor	11.02 mm ± 2.4	15.10 mm ± 1.9	0.01*	13.16 mm ± 2.0	17.10 mm ± 1.8	0.01*
Upper molar	12.57 mm ± 2.7	13.69 mm ± 2.2	0.05*	7.32 mm ± 1.6	8.80 mm ± 2.5	0.03*
Lower molar	15.98 mm ± 4.4	11.7 mm ± 1.9	0.001**	9.70 mm ± 2.6	6.30 mm ± 1.05	0.002**

*Significant level (*p* < 0.05), **Significant level (*P* < 0.01).

**Table 3:** Independent sample t-test between the pre-pubertal and post-pubertal CBCT treatment changes.

Variable	Pre- pubertal n = 18 T_2_–T_1_	Post- pubertal n = 18 T_2_–T_1_	*P* value
	Mean ± SD	Mean ± SD	
SNA	− 0.22° ± 0.05	− 0.64° ± 0.09	0.4
SNB	1.53° ± 0.04	.3° ± 0.7	0.02*
Point A	− 0.30 mm ± 0.04	− 0.29 mm ± 0.09	0.9
Point B	2.13 mm ± 1.7	.52 mm ± 0.9	0.03*
Glenoid fossa	0.86 mm ± 0.06	0.17 mm ± 0.02	0.3
Upper incisor	(−) 1.03 mm ± 0.1	(−) 1.48 mm ± 0.6	0.5
Lower incisor	(+) 4.08 mm ± 0.3	(+) 3.93 mm ± 0.2	0.8
Upper molar	(−) 1.12 mm ± 0.5	(−) 1.49 mm ± 0.6	0.6
Lower molar	(+) 3.60 mm ± 0.1	(+) 2.89 mm ± 0.7	0.4

Significant (*p* < 0.05).

In the incisors and molar position, the (+) sign indicates mesial movement, (−) sign indicates distal movement.

The skeletal effect of Herbst treatment on the mandibular forward position was assessed by linear sagittal measurements of point B and SNB angle. In the Pre-pubertal group, there was a statistically significant increase in both measurements (point B: 2.13 mm ± 1.7, *P* < 0.01; SNB angle: 1.53º ± 0.04, *P* = 0.01). On the contrary, the Post-pubertal group showed statistically non-significant skeletal treatment effects on the mandible (point B: 0.52 mm ± 0.9, *P* > 0.05; SNB angle: 0.3º ± 0.7, *P* > 0.05). No maxillary skeletal effect was found in the Pre-pubertal group, as evident from the position of point A (− 0.3 mm ± 0.04, *P* = 0.4) and SNA angle (− 0.22º ± 0.05, *P* = 0.6); similar results were also observed in the Post-pubertal group where there were no statistically significant changes in both measurements (point A: − 0.29 mm ± 0.09, *P* = 0.3; SNA angle: − 0.64º ± 0.09,* P* = 0.07) (Table 1).

When comparing the treatment results between the pre- and Post-pubertal groups, both the sagittal mandibular readings, SNB angle (*P* = 0.03), and the horizontal position of point B (*P* = 0.03) exhibited statistically significant differences. However, there were no statistically significant differences for any maxillary skeletal measurements between both groups (*P* > 0.05; Table 2). The condylar-head position did not show any significant change after Herbst treatment in both groups (Tables 3, 4).

**Table 4:** Pair-wise t-test between the pre and post treatment TMJ readings as shown in the CBCT in the pre and post-pubertal group.

Variable	Pre- pubertal group			Post- pubertal group		
	Pre- treatment (T1) Mean ± SD	Post-treatment (T2) Mean ± SD	*P*	Pre- treatment (T1) Mean ± SD	Post-treatment (T2) Mean ± SD	*P*
Anterosuperior space (R) (mm)	2.382 ± 0.4	2.371 ± 0.6	0.9	2.294 ± 0.5	2.133 ± 0.7	0.5
Anterosuperior space (L) (mm)	2.394 ± 0.5	1.985 ± 0.7	0.1	2.503 ± 0.3	2.338 ± 0.4	0.3
Superior space (R) (mm)	2.594 ± 0.4	2.871 ± 0.8	0.3	3.317 ± 0.8	3.084 ± 0.8	0.2
Superior space (L) (mm)	2.664 ± 0.4	2.792 ± 0.8	0.6	3.192 ± 1.0	3.26 ± 0.5	0.7
Posterosuperior space (R) (mm)	2.257 ± 0.4	2.689 ± 0.9	0.07	2.94 ± 0.7	3.371 ± 1.0	0.1
Posterosuperior space (L) (mm)	2.599 ± 0.6	2.854 ± 0.9	0.5	3.165 ± 0.9	3.31 ± 0.9	0.7
Posterior space (R) (mm)	2.894 ± 1.0	3.136 ± 1.1	0.2	3.985 ± 1.0	3.803 ± 1.4	0.5
Posterior space (L) (mm)	2.896 ± 0.7	3.208 ± 1.0	0.3	3.531 ± 1.4	3.803 ± 1.0	0.2
Anterosuperior space (R) (mm)	2.382 ± 0.4	2.371 ± 0.6	0.9	2.294 ± 0.5	2.133 ± 0.7	0.5
Condylar access/midsagittal plane (R, angle) (°)	66.07 ± 3.6	65.5 ± 3.4	0.7	66.64 ± 4.6	68.39 ± 4.0	0.2
Condylar access/midsagittal plane (L, angle) (°)	67.87 ± 4.9	69.45 ± 3.2	0.2	68.31 ± 4.5	66.59 ± 4.6	0.4
Condylar access/midsagittal plane (R, distance) (mm)	58.62 ± 2.9	58.02 ± 2.7	0.6	58.68 ± 8.0	59.53 ± 4.6	0.5
Condylar access/midsagittal plane (L, distance) (mm)	60.04 ± 5.7	60.55 ± 4.6	0.7	58.86 ± 3.6	58.5 ± 4.4	0.7

Significant level (*p* < 0.05).

**Table 5:** Independent sample t-test between the pre-pubertal and post-pubertal treatment changes of the TMJ readings as shown in the CBCT.

Variable	Pre- pubertal n = 10 T2–T1	Post- pubertal n = 10 T2–T1	*P* value
	Mean ± SD	Mean ± SD	
Anterosuperior space (R) (mm)	− 0.010 ± 0.7	− 0.16 ± 0.8	0.6
Anterosuperior space (L) (mm)	− 0.40 ± 0.8	− 0.16 ± 0.4	0.4
Superior space (R) (mm)	0.27 ± 0.9	− 0.23 ± 0.6	0.1
Superior space (L) (mm)	0.12 ± 0.9	0.067 ± 0.7	0.8
Posterosuperior space (R) (mm)	0.42 ± 0.8	0.43 ± 0.6	0.9
Posterosuperior space (L) (mm)	0.25 ± 1.1	0.14 ± 1.3	0.8
Posterior space (R) (mm)	− 0.24 ± 0.8	0.18 ± 0.5	0.2
Posterior space (L) (mm)	0.31 ± 0.9	0.27 ± 0.7	0.9
Condylar access/midsagittal plane (R, angle) (°)	− 0.5 ± 5.1	1.75 ± 4.9	0.3
Condylar access/midsagittal plane (L, angle) (°)	1.58 ± 3.7	− 1.72 ± 7	0.2
Condylar access/midsagittal plane (R, distance) (mm)	− 0.6 ± 4.4	0.85 ± 4.6	0.4
Condylar access/midsagittal plane (L, distance) (mm)	0.51 ± 4.4	− 0.36 ± 3.5	0.6

Significant level (*p* < 0.05).

## Discussion

The skeletal and dental effect of Herbst appliance has been investigated mostly by the conventional cephalometric radiographic techniques. Using those techniques, the researchers usually suffer from difficulty in identifying some landmarks such condylar head and glenoid fossa. The 2-dimensional radiographic image is subjected to magnification, distortion, patient positioning errors, and obstruction of critical landmarks by overlapping anatomic structures. The 2-dimensional linear and angular cephalometric measurements provide limited information’s about the movements of the structures and do not explain the complex 3-dimensional process of dento-skeletal response to the treatment. Additionally, the inherent examiner bias in the registration and superimposition process if the examiners are not blinded brings to question of the accuracy concerning the 2D readings.

CBCT was developed from computed tomography (CT) and adapted to the use in the maxillofacial region, it has lower operational costs, more compact equipment and radiation dosage is much lower when compared to spiral C.T which frequently used in the field of medical imaging. At the same time CBCT offers images with corrected magnification and almost without distortion, also identifying the land marks is much accurate because of the ability to visualize the structures and perform measurements in specific axial, coronal, and sagittal slices to evaluate a specific area of interest. The main disadvantage of the CBCT in the present study is lack of norm for the study population.

Therefore, the bottom line of the present study was to investigate the changes after Herbst appliance treatment and relate them to the pubertal status of the skeletal Class II patients using CBCT. The pubertal status was determined with respect to the CVMS by Bacetti et al.^8^. This method utilizes data derived from 3 cervical vertebrae: C2, C3 and C4, as visualized in a the lateral cephalogram. Five maturational stages (CVMS I through CVMS V) with the peak in mandibular growth occurring between CVMS II and CVMS III. The peak in mandibular growth will occur not earlier than one year after stage CVMS I, and within one year after stage CVMS II. Stages CVMS IV and CVMS V are considered postpubertal where the peak in mandibular growth has occurred not later than one year before stage CVMS IV, and two years before stage CVMS V. Stage CVMS III considered transitional stage and excluded from the study^8^.

### Skeletal and dental changes

Previous reports on the maxillary restraining effect are controversial; in the present study, there were no statistically significant changes in any of the two variables used to evaluate the maxillary impact for both age groups. The insignificant effect on the maxilla is in agreement with those of previous studies^9,10^. However, this decrease is not of statistical significance, yet it could be of clinical importance, particularly in growing patients, because it suggests restriction of the maxillary growth that is expected to take place on this period for Class II patients^11^.

However, other studies reported that Herbst had a significant restraining effect on maxillary growth^3,12^. This controversy could be explained by variation in treatment age, different treatment mechanics, or treatment duration. The reported decrease in SNA could not be exclusively attributed to the limitation of maxillary growth; it could be due to the remodeling at A-point due to the distal tipping of the incisors.

According to the result of the present study, the Herbst IV appliance resulted in significant retroclination of the upper incisors and distalization of the upper molars, which could be the result of the distally directed force of the appliance (headgear effect) on the maxillary arch. This result is in agreement with previous studies that have reported similar findings^13–16^.

Since most Class II patients come from mandibular rather than maxillary origin^17^, the question about those patients' ability to catch up mandibular growth in two different maturity stages using Herbst appliances is the cornerstone of the present study.

After Pancherz reintroduced the Herbst in 1979^18^ broad debate was raised about the possible effect of the Herbst appliance on the mandible in patients with limited growth potential. He published a study in 1997 and reported a non-significant difference between 3.1 mm sagittal advancement in the early treatment (defined by maturity stages MP3-E and F) and 2.4 mm in the late treatment (defined maturity stages MP3-H and -I). Two years later^19^, he reported significant difference of 2.3 mm for young adults (defined R-IJ and RJ) and 4.3 mm for adolescents (defined byMP3-E to MP3-G). Later^20^, he compared the effect of Herbst appliance treatment and sagittal split osteotomy on mandibular base advancement and showed that the latter had a more significant impact than Herbst appliance. In the present study, the pre-pubertal group showed a significant increase in SNB as well as forward displacement of point B. On the contrary, mandibular sagittal position in Post-pubertal group exhibited a non-significant increase, indicating that the Herbst could not stimulate forward mandibular growth. This finding agrees with those of other studies that reported little or no effect on mandibular growth on long term^21,22^. The results of the present study emphasize the importance of detection and treatment of Class II cases in the Pre-pubertal years to get more skeletal and stable results, while the results in the post-pubertal years are nearly dental.

Forward movement of the lower molars and proclination of the lower incisors were also evident. Weschler and Pancherz^14^ reported, "Mandibular anchorage loss in Herbst treatment is a reality with which the orthodontist has to live with" Lower incisor inclination changes in both groups demonstrated significant proclination, which was an inevitable side effect of Herbst with subsequent early correction of the overjet, thus limiting skeletal correction^1–3,6^. Clinicians should be aware of that when planning Herbst treatment for Class II cases with initially proclined lower incisors by adding TADs to support the lower incisors. Also, selecting brackets with negative crown toque in the second stage of the treatment could be helpful.

### Condyle-fossa relationship changes

One of the questions addressed in the current CBCT study was the condylar heads' ability to return to their original position in the glenoid fossa after 7 months of bite jumping using Herbst appliance. When the mandible is jumped to the desired sagittal therapeutic position, the condyles are expected to be out of centric relationship and move ventrally from the natural position within the mandibular fossa into the direction of the articular tuberculum^23^. The establishment of a proper condylar position is one of the most important factors in postoperative stability. The non-significant change in the joint space observed in the present study of both age groups could be explained by the adaptation and remodeling of the condyles approximately to their initial positions after seven months of bite jumping. In our study, the sagittal position of the right and left glenoid fossa did not change significantly after using Herbst IV appliance in both groups. This result did not support the claim that the glenoid fossa relocation in inferior and anterior directions plays a significant role in Class II correction^24–26^. In another study, the position of condyle within the fossa had no significant change as the findings of the present study, while there was a deposition at posterior wall of glenoid fossa and simultaneous remodelling changes occurring in condyle resulting in growth stimulation in Herbst treated subjects^27^.

Another study^28^ showed that posterior condylar point was 0.96 mm more posteriorly and a superior condylar point 2.27 mm more superiorly when compared with a single-phase regime with Class II elastics, the results of this study derived from 3D mandibular regional voxel-based suerperimposition technique not positional changes.

### Limitations

Gender distribution, as well as the lack of CBCT data for matching non-treatment controls, are limitations of this study. Future studies may utilize matching control CBCT to eliminate possible growth effects on the Herbst treatment outcome.

## Conclusions

- Seven months of treatment with Herbst IV appliance corrected dental Class II in both groups to Class I molar relationships. However, the mandibular skeletal effect was more evident in the younger age group with more stable results and less tendency for relapse, emphasizing the importance of early detection and treatment of skeletal Class II.
- The non-significance difference in condylar position within the TMJ fossa in both groups suggests that TMJ remodeling has occurred in both groups.

## Methods

### Trial design and any changes after trial commencement

This study was a single-center randomized clinical trial performed in in the Outpatient Clinic, Orthodontic Department, Faculty of Dental Medicine, Al-Azhar University, Cairo (Boys). No changes occurred during the trial. (ClinicalTrials.gov identifier: NCT04518865-19\8\2020).

### Participants, eligibility criteria, and settings

The study design has been approved by the Research Ethics Committee of Orthodontic Department, Faculty of Dentistry, Al-Azhar University, Egypt. Informed consent from all subjects and/or their legal guardian(s) for publication of identifying information/images in an online open-access publication and for the participation in the present study were obtained. The study was performed in accordance with the relevant guidelines and regulations.

### Inclusion criteria

- Skeletal Class II malocclusion with retrognathic mandible (ANB > 5°, SNB < 76°)
- Regular growth pattern (SN/MP angle was in 25–35° range)
- Unilateral or bilateral Class II molar relationship greater than or equal to one-half a cusp width.
- Minimal or no crowding in the mandibular arch (0–5 mm)
- An average inclination or slight retroclination of the lower incisor
- An overjet greater than 5 mm.

### Sample size calculation

The sample size calculation was based on the feasibility of detecting a clinically meaningful difference in mandibular length of 2.3 mm (± 1.7 mm), with an alpha error of 0.05 and a test power of 80%^29^. The calculation was performed using the G Power software (Universität Düsseldorf, Germany). Accordingly, the recommended sample size was 16 patients in each group. To compensate for a possible dropout during the study period, 18 patients were included in each group.

### Randomization

Patients were randomly selected from the prescreening tool that has been used at the University using a computer-generated random list. The patients were allocated into two parallel groups; Pre-pubertal growth and post-pubertal (with allocation ratio 1:1) based on their skeletal stage of maturation using the modified cervical vertebrae maturation staging (CVMS) by Bacetti et al.^8^. Pre-pubertal group: (N = 18; 8 females and 10 males) with mean age 9.15 ± 1.5 years, with skeletal maturity stages of CVMS I and CVMS II. Post-pubertal group: (N = 18; 9 females and 9 males) with mean age 16.3 ± 1.0 years, with skeletal maturity stages CVMS IV and CVMS V.

### Interventions

**Figure 1:**
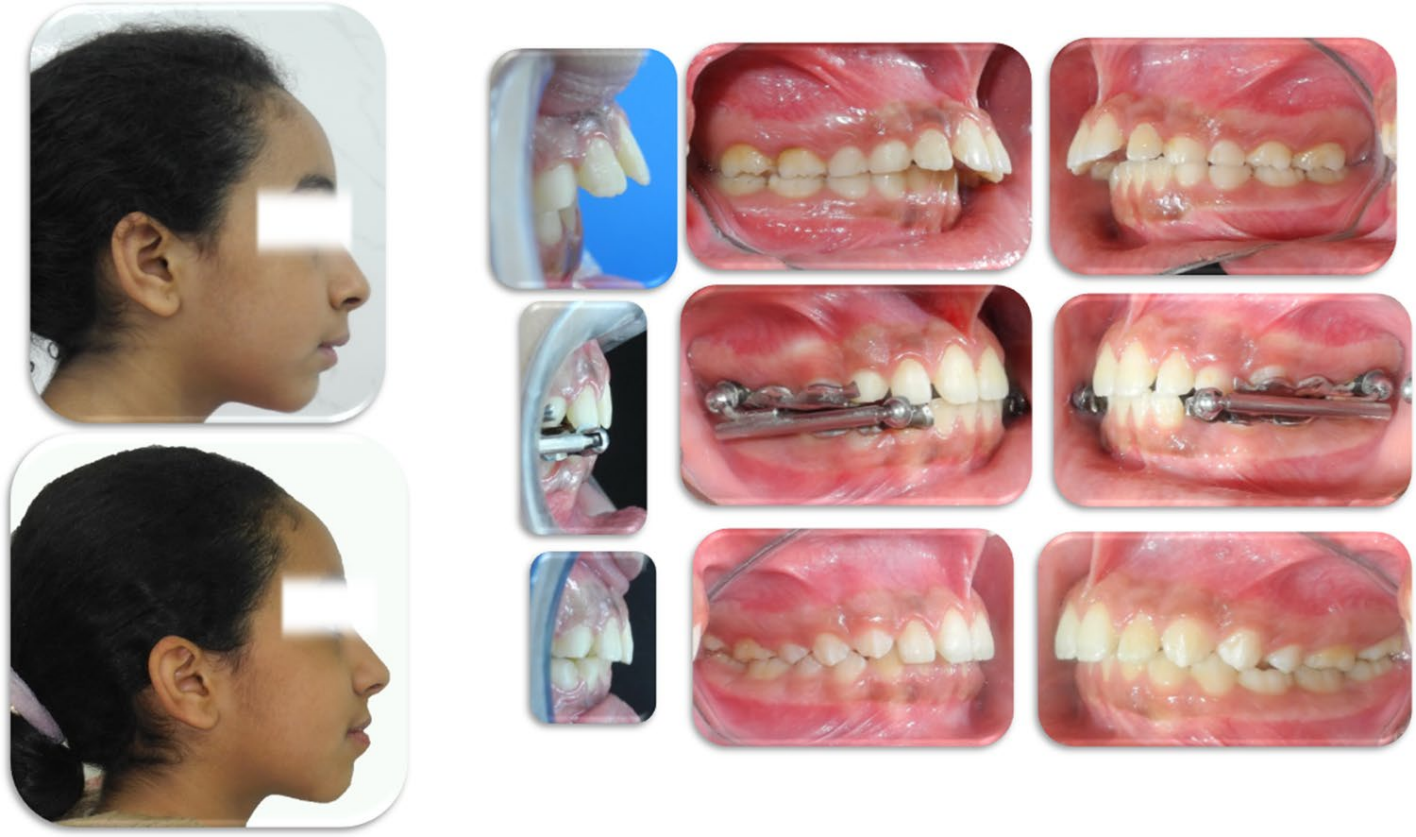
Pre-pubertal Class II case; before, during, and after Herbst IV treatment.

**Figure 2:**
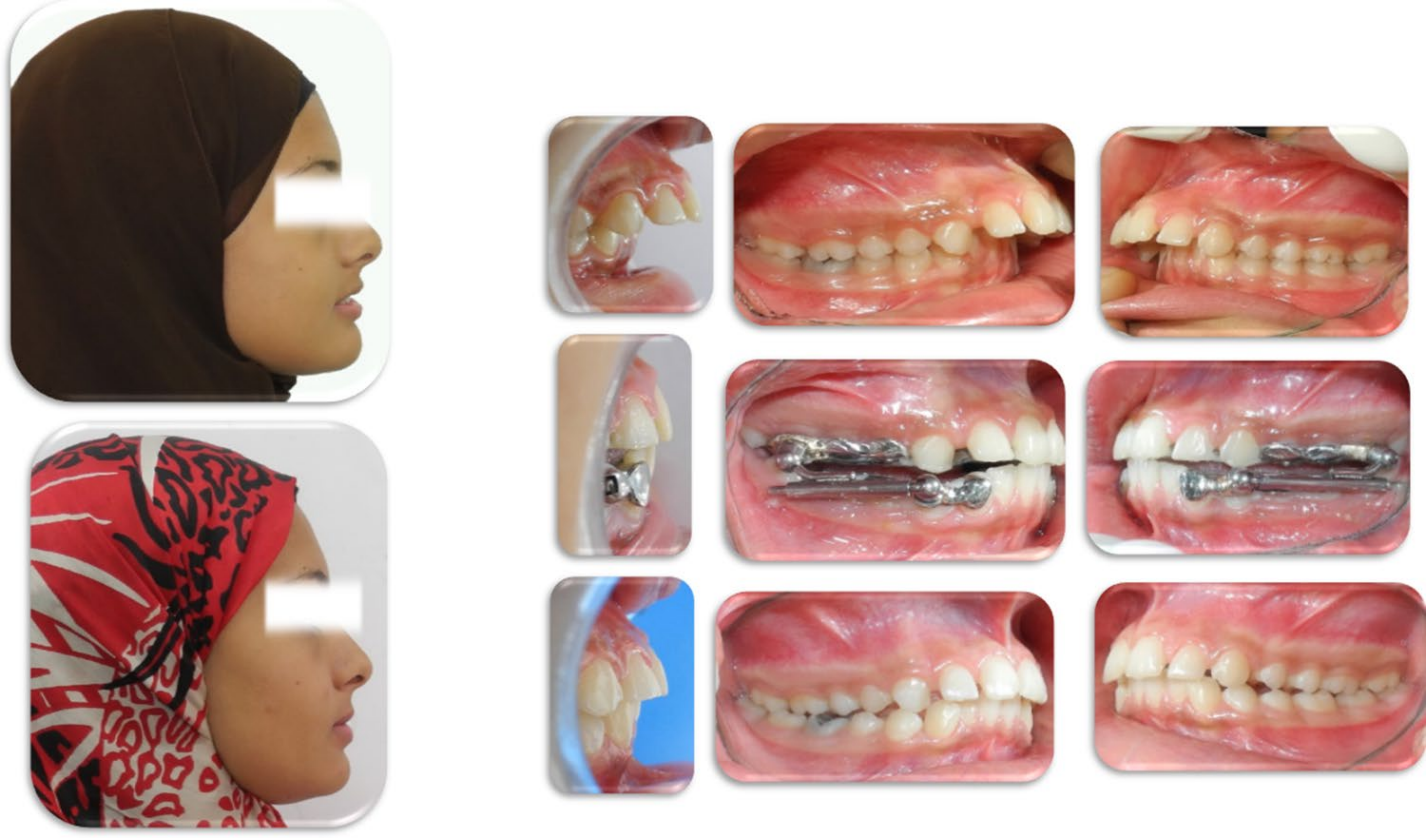
Post-pubertal Class II case; before, during, and after Herbst IV treatment.

All patients in both groups were treated with the metallic splint-supported Herbst IV appliance (Dentaurum GmbH & Co. Germany; Figs. 1, 2). In the maxillary arch, the splints covered the premolars or the deciduous molars and first molars with a palatal plate touching the palatal surface of the incisors. In the mandibular arch, the splints covered the canines, premolars, or deciduous molars, and first molars with a lingual plate touching the lingual surface of the incisors. Treatment time averaged 7 months. The Herbst appliance was activated initially to an edge-to-edge incisor relationship. Cone-beam computed tomography CBCT (Scanora 3D, Soredex, Finland). The CBCT scanning setting was as follows: FOV: 13 × 14.5 cm, Voxel size (mm): 0.35, Scan/ Exposure time (s): 20/4.5, Total image processing time approx. (minutes): 2, Receptor type: CMOs Flat Panel, Receptor active area: 124 mm × 124 mm, Pixel size: 200 µm), scans were taken before and after treatment.

### Outcomes

The primary outcomes of the study were sagittal skeletal and dental changes. The secondary outcomes were condylar head position within the TMJ/fossa.

### CBCT imaging and 3D analysis

#### Skeletal and dental changes

**Figure 3:**
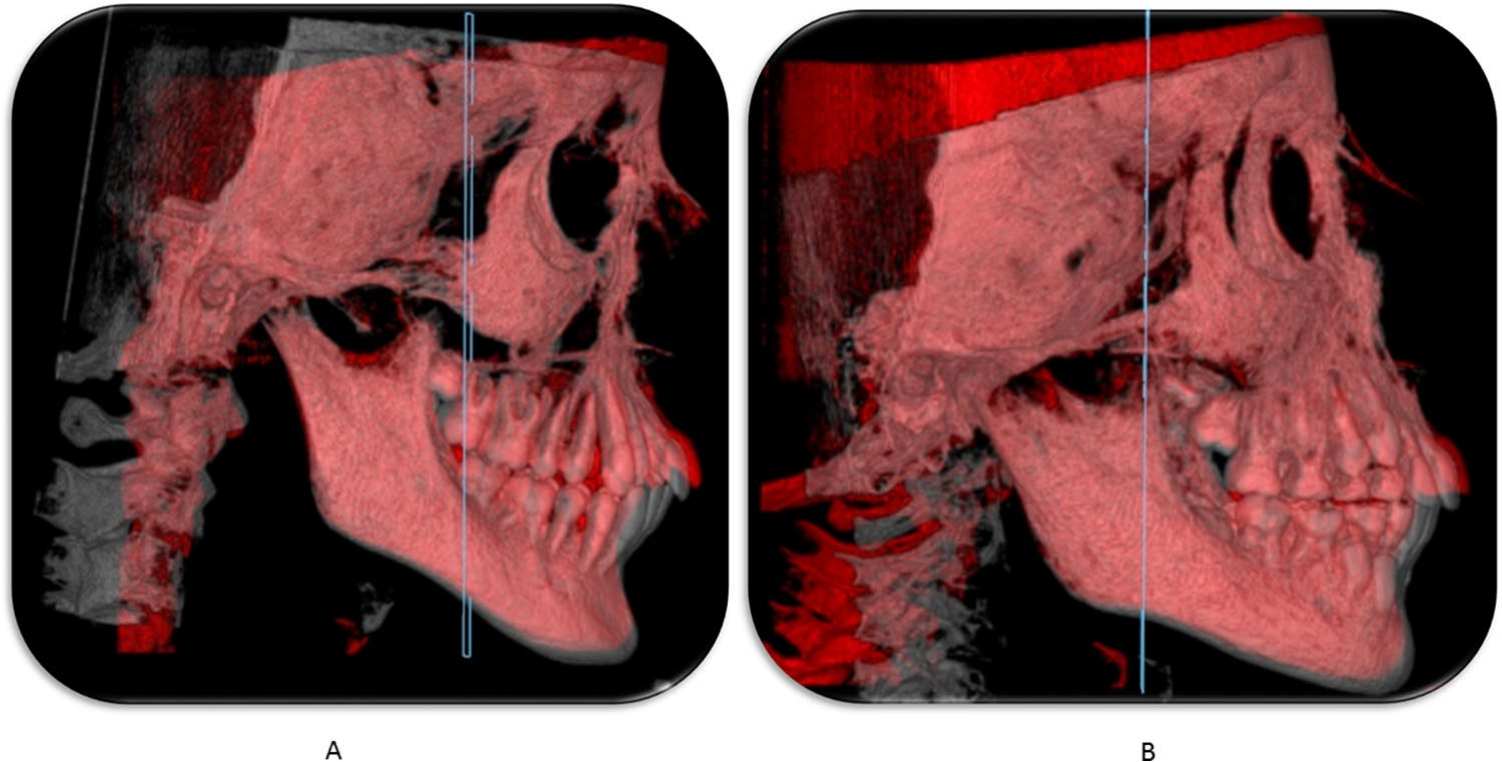
3D view for Herbst treatment; (**A**) pre-pubertal (**B**) Post-pubertal.

**Figure 4:**
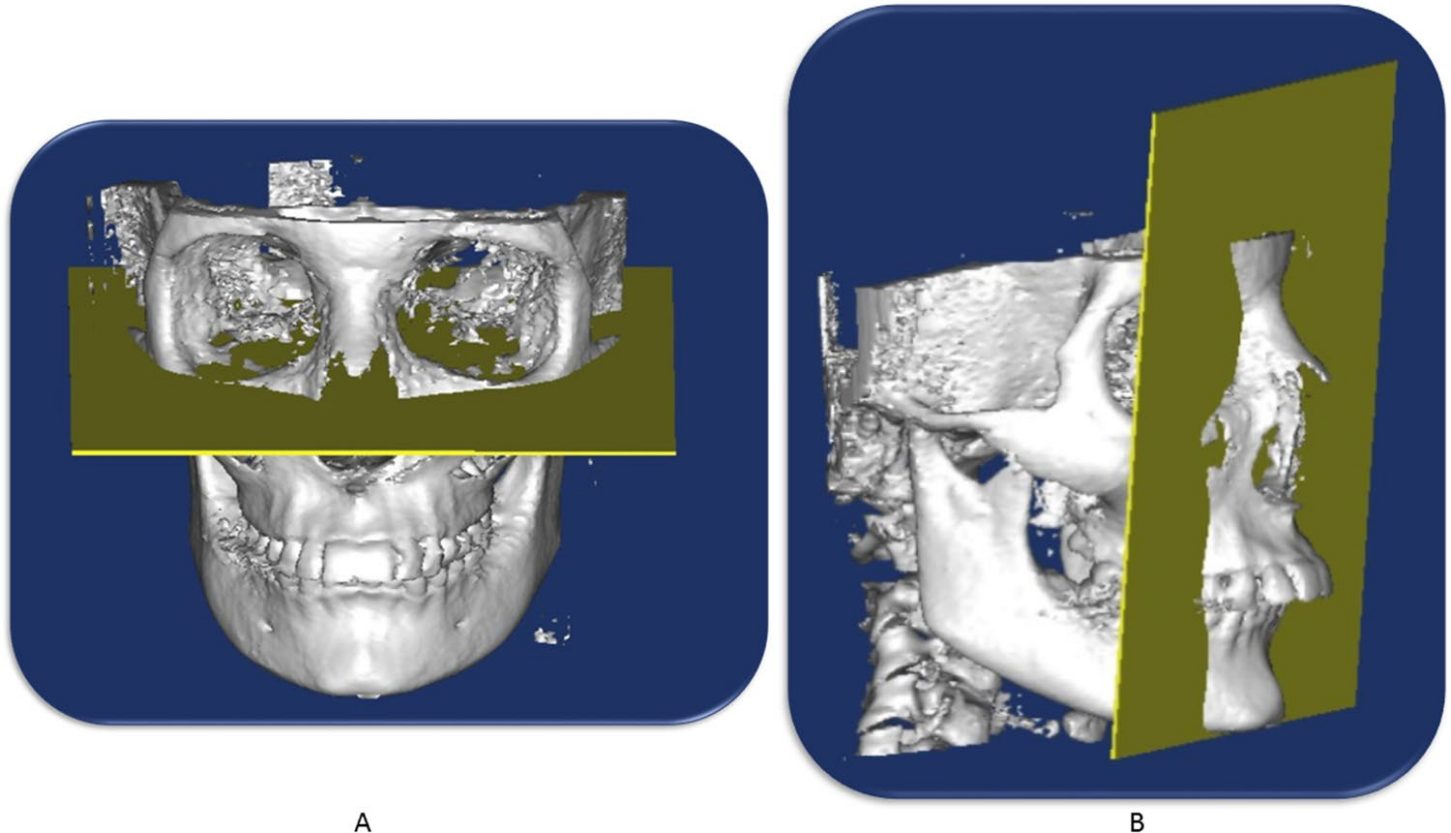
Frankfort horizontal plane(**A**), and orbital perpendicular plane (**B**).

**Figure 5:**
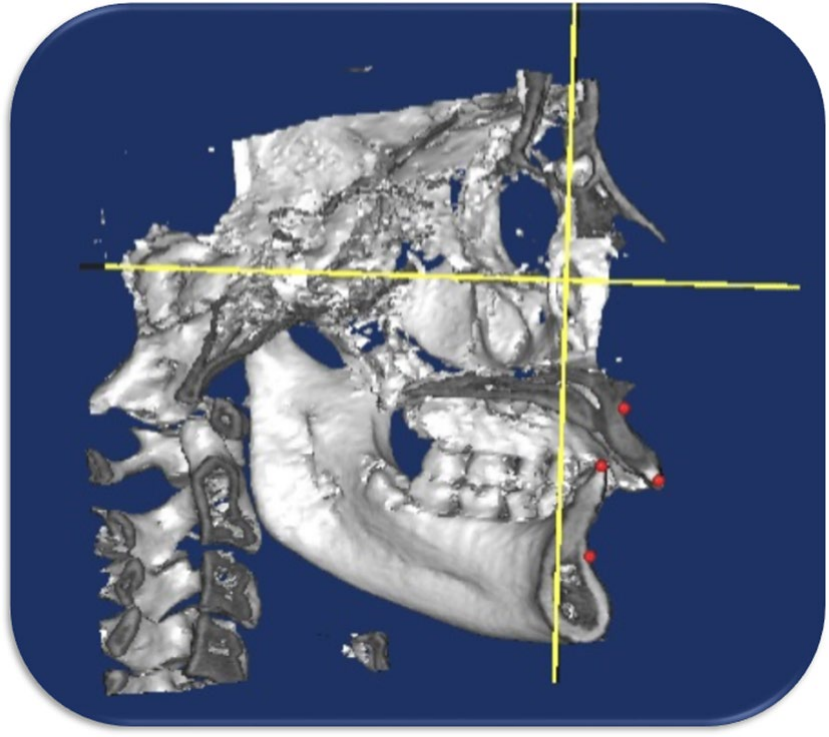
The sagittal relation for point A, point B, upper incisor, and lower incisor to the orbital perpendicular plane.

The DICOM (Digital Imaging and Communications in Medicine) files from the pre and post-CBCT (Fig. 3) scans were imported to Mimics software (Version 10.1, Materialise NV, Leuven, Belgium); and the cephalometric landmarks and planes were defined (Figs. 4, 5) (Table 5). Linear sagittal measurements of point A, point B, glenoid fossa, upper incisor, lower incisor, upper molars, and lower molars were recorded in relation to the orbital perpendicular plane.

#### Temporomandibular joint changes

**Figure 6:**
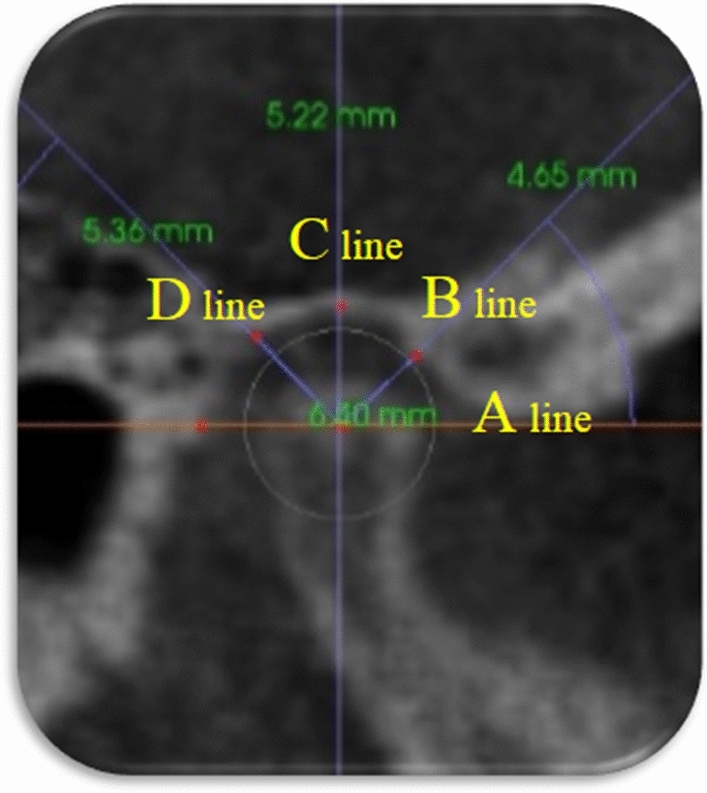
Horizontal line was placed through the widest width of the condylar head (A), three lines crossing the glenoid fossa at 45°(B), 90°(C), and 135° (D).

**Figure 7:**
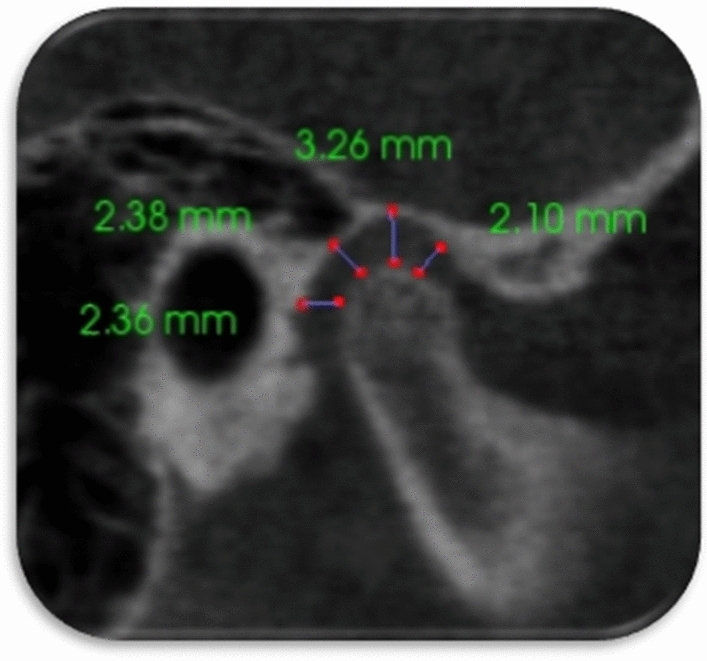
Anterosuperior, superior, posterosuperior, and posterior TMJ spaces.

To analyze the condylar head positional changes within the glenoid fossa; the most middle-superior point of the mandibular condyle was determined in sagittal, axial, and coronal views^30^. In the sagittal slice of the middle condylar head, a line was drowned through the broadest measurement of the condylar heads visible on the slice; from this line, three lines were raised crossing the glenoid fossa at 45°, 90°, and 135°^31^ (Fig. 6). Four linear measurements representing the anterosuperior, superior, posterosuperior, and posterior distance were taken from the glenoid fossa wall to the surface of the condyle (Fig. 7).

**Figure 8:**
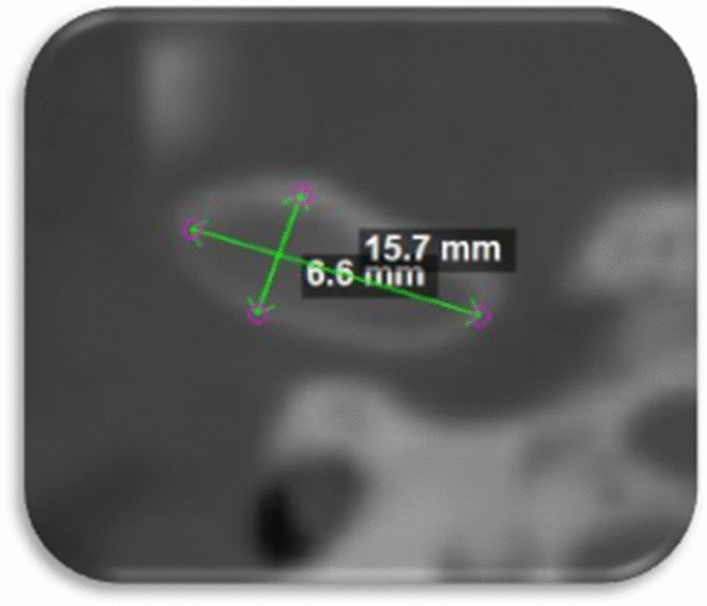
The geometric center of the condylar head as the point of intersection between the greatest anteroposterior and mediolateral diameter of the condylar process.

And to assess the lateral changes of the condylar head; the geometric center of the condylar head was determined in the axial slice as the point of intersection between the greatest anteroposterior and mediolateral diameter of the condylar process (Fig. 8).

**Figure 9:**
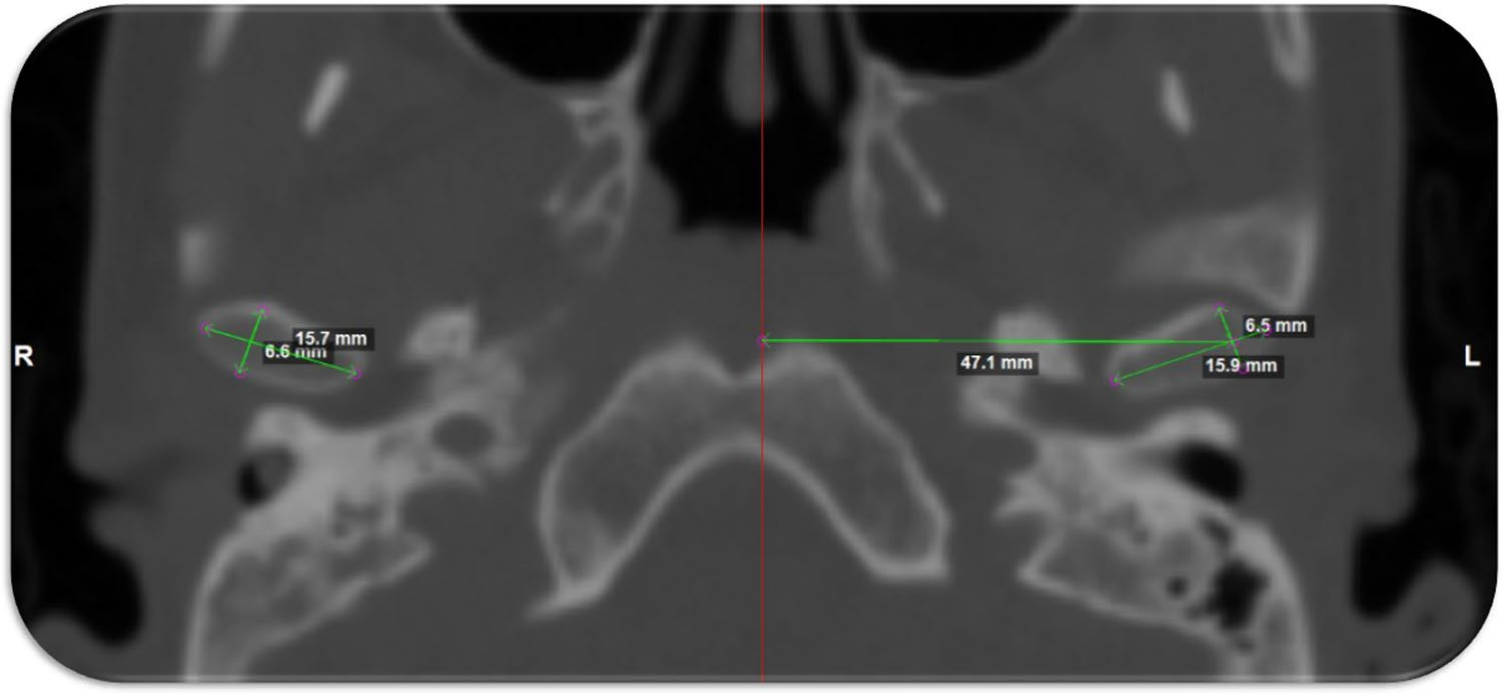
The lateral distance between the geometric center of the condylar processes to the midsagittal plane.

**Figure 10:**
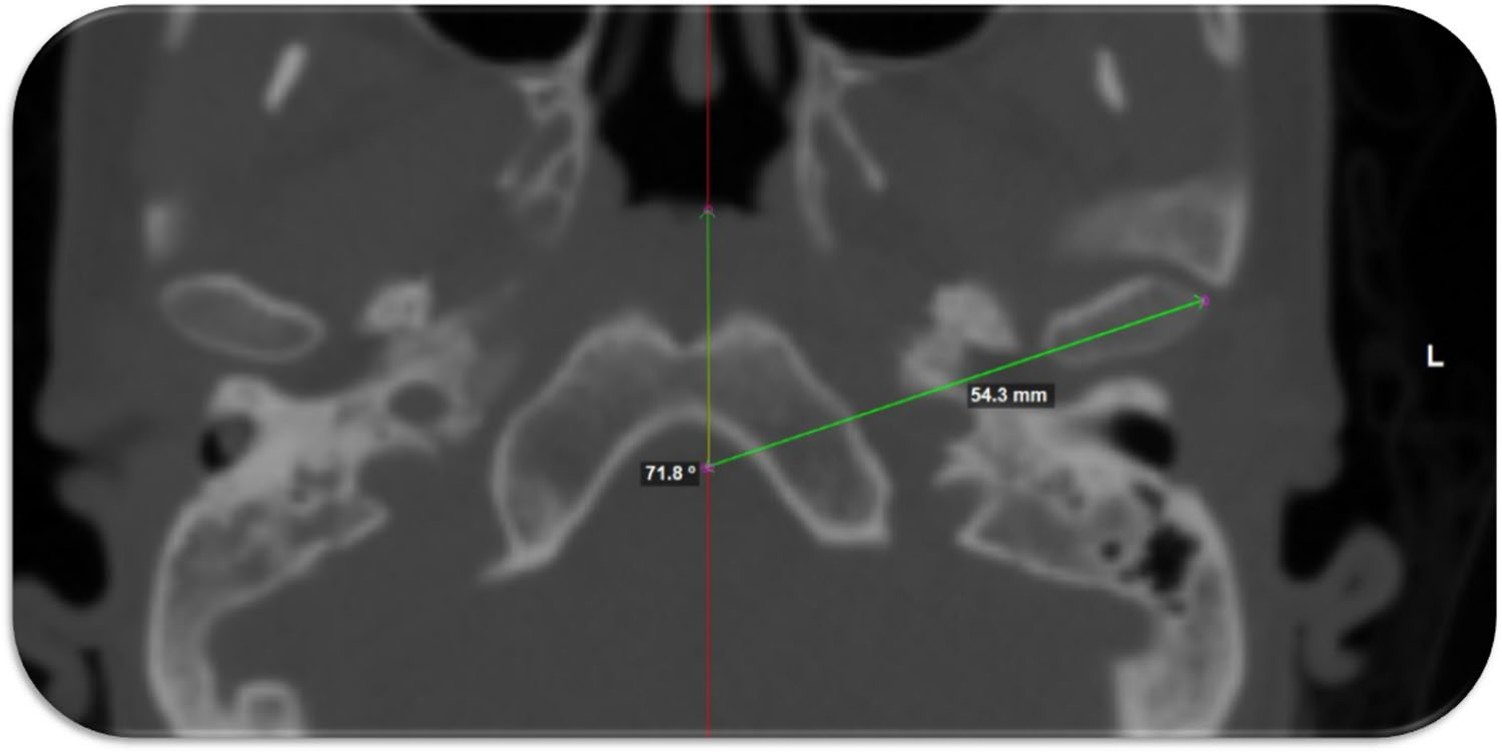
The angel between the condylar long access of the mandibular condyle and the midsagittal plane.

The linear distance between the geometric center to the midsagittal plane was measured (Fig. 9). The lateral orientation was assessed by measuring the angel between the condylar long access and the midsagittal plane (Fig. 10)^32^.

### Reliability

To assess the error in locating the points, 20 CBCT scans were randomly selected and remeasured by the same examiner after one month. Dahlberg’s formula was used to measure the casual error, and the result did not exceed 0.9 mm and 1.1º. Paired t-tests demonstrated no statistically significant systematic error differences for all measurements.

### Blinding

Blinding of both the clinician and patient to the intervention was impossible. However, the clinician who analyzed the CBCT measurements were blinded regarding the groups to which the participants belonged. All data were labeled with codes and were transferred to a statistician who was also blinded regarding the patients’ groups.

### Statistical analysis

Statistical analysis was performed in SPSS Version 20.0 (SPSS Inc, Chicago, Ill). The mean and standard deviation values were calculated for each group. Data were tested for normality using Kolmogorov–Smirnov and Shapiro–Wilk tests and showed normal distribution. Therefore, Pair-wise *t*-test was used to compare different variables (pretreatment T1 and post-treatment T2) within the same group, and independent t-test was used to compare pre-pubertal and post-pubertal groups. The significance level was set at *P* ≤ 0.05.

## Data Availability

The data that support the findings of this study are available from the corresponding author upon reasonable request.
